# Perianal atypical leiomyoma: A case report: Erratum

**DOI:** 10.1097/MD.0000000000010313

**Published:** 2018-03-30

In the article, “Perianal atypical leiomyoma: A case report”,^[1]^ which appeared in Volume 96, Issue 48 of *Medicine*, affiliation a appeared incorrectly and should appear as:

Department of Anorectal Surgery, The First Affiliated Hospital of Guangxi Medical University, Nanning, China.
